# Persistence of SARS-CoV-2 Alpha Variant in White-Tailed Deer, Ohio, USA

**DOI:** 10.3201/eid3107.241922

**Published:** 2025-07

**Authors:** Natalie N. Tarbuck, Sofya K. Garushyants, Dillon S. McBride, Patricia M. Dennis, John Franks, Karlie Woodard, Austin Shamblin, Michael G. Sovic, Derek T. Collins, Kyle Van Why, Richard J. Webby, Martha I. Nelson, Andrew S. Bowman

**Affiliations:** The Ohio State University, Columbus, Ohio, USA (N.N. Tarbuck, D.S. McBride, P.M. Dennis, A. Shamblin, M.G. Sovic, A.S. Bowman); National Institutes of Health, Bethesda, Maryland, USA (S.K. Garushyants, M.I. Nelson); Cleveland Metroparks Zoo, Cleveland, Ohio, USA (P.M. Dennis); St. Jude Children's Research Hospital, Memphis, Tennessee, USA (J. Franks, K. Woodard, R.J. Webby); Animal and Plant Health Inspection Service, Fort Collins, Colorado, USA (D.T. Collins); Animal and Plant Health Inspection Service, Harrisburg, Pennsylvania, USA (K. Van Why).

**Keywords:** SARS-CoV-2, white-tailed deer, public health, animal reservoir, viruses, zoonoses, Ohio, United States

## Abstract

Free-ranging white-tailed deer (WTD) are highly susceptible to the SARS-CoV-2 virus. Through an opportunistic sampling of WTD in northeast Ohio, USA, during January–March 2023, we identified 6 SARS-CoV-2 lineages from 36 sequences using the pangolin lineages tool, including the B.1.1.7 lineage (Alpha variant) and BQ.1.1, BQ.1.1.63, BQ.1.1.67, BQ.1.23, and XBB.1.5.35 lineages (Omicron variant). The Alpha variant, introduced by a single human-to-deer transmission event, was detected in 5 WTD in January 2023, more than 1 year after the most recent detection of the Alpha variant in humans in Ohio (August 2021). A genetically similar B.1.1.7 lineage virus from WTD in a nearby county in Pennsylvania was positioned with our Ohio deer transmission cluster, suggesting deer-to-deer transmission. The persistence of the Alpha variant in WTD in Ohio warrants continued surveillance to monitor if WTD can become a reservoir for displaced SARS-CoV-2 variants.

Since the beginning of the COVID-19 pandemic, SARS-CoV-2 evolution has produced multiple variants that affected public health by causing new epidemics, replacing prior variants, and evading existing immunity. SARS-CoV-2 is primarily transmitted from human to human, although human-to-animal transmission is documented in a wide variety of animal species (*1*). The role of host-switching in viral evolution of SARS-CoV-2 is not fully understood, yet some speculate that the emergence of the Omicron variant may be a result of animal-acquired mutations (*2*,*3*)

Free-ranging white-tailed deer (WTD) are susceptible to the SARS-CoV-2 virus, raising concerns that they might become a new animal reservoir for a diverse viral population or sustain older variants displaced in humans (*4*). SARS-CoV-2 infection in WTD was reported in northeast Ohio, USA, in 2021 (*5*) and the virus has since been detected in WTD across North America (*4*,*6*–*10*). SARS-CoV-2 evolves ≈3 times faster in deer than in humans, which makes evolutionary divergence possible (*11*). Of note, a highly divergent SARS-CoV-2 lineage, B.1.641, was linked to suspected deer-to-human transmission (*8*), emphasizing the risk associated with emergence of a deer-adapted virus in human populations.

There are ≈30 million WTD in the United States, and population levels pose ecologic and human safety challenges as hunting rates decline (*12*,*13*). Interactions between deer and humans create pathways for zoonotic transmission of SARS-CoV-2, yet the mechanisms driving this transmission remain unclear. If WTD become a natural reservoir for SARS-CoV-2, there is potential for deer-lineage and human-lineage viruses to diverge over time. To monitor the persistence and evolution of SARS-CoV-2 and define mutations and mutation rates in WTD, we conducted SARS-CoV-2 surveillance in northeast Ohio, an area with a historically high prevalence of SARS-CoV-2 in WTD.

## Methods

### Sample Collection

We collected 519 nasal swab specimens from free-ranging WTD that were culled during management activities in northeast Ohio during January–March 2023. The WTD were from 10 study sites covering ≈1,000 km^2^ of landscape of varying human population density where hunting is not permitted. We collected nasal swab specimens from each fresh deer carcass by using a sterile polyester tipped swab then placed into a 3-mL vial containing viral transport medium (VTM). We chilled samples in the field and stored them at −80°C until testing. Sample collection was conducted after death and was exempt from oversight by The Ohio State University Institutional Animal Care and Use Committee.

### Diagnostic Testing

We tested all collected samples for SARS-CoV-2 RNA by using quantitative real-time reverse transcription PCR (RT-PCR). We extracted viral RNA from 200 μL of VTM by using the MagMAX Viral/Pathogen II Nucleic Acid Isolation Kit (Thermo Fisher Scientific, https://www.thermofisher.com). We tested the extracted viral RNA from each sample with quantitative real-time RT-PCR by using the TaqPath COVID-19 Combo Kit with MS2 phage control (Thermo Fisher Scientific). We considered any sample with a cycle threshold (Ct) value of <37 on >2 targets (nucleocapsid gene, spike gene, open reading frame 1ab) SARS-CoV-2 positive. We sent samples with a Ct <33 for whole-genome sequencing.

### Genomic Sequencing

We reverse transcribed extracted RNA into cDNA by using Lunascript RT Supermix (New England Biolabs, https://www.neb.com). After transcription, we divided the sample in half and separately enriched for SARS-CoV-2 with 35 cycles of PCR by using the ARTIC 4.1 primer sets and Q5 Hot Start (New England Biolabs). We then recombined the enriched products and conducted a 1.8× cleanup by using AmpureXP beads (Beckman Coulter, https://www.beckman.com) and resuspended in resuspension buffer (Illumina, https://www.illumina.com). We conducted tagmentation with enrichment beads according to the Illumina DNA Prep protocol (Illumina) with unique dual indexes. We pooled individual libraries in equimolar amounts and performed 2 × 150 bp sequencing on an Illumina NextSeq2000 (Illumina). We generated fastq files by using BCL Convert and ran the files through DRAGEN Covid Lineage v3.5.1 on Basespace (Illumina). We filtered the fastq files with sequencing reads by using fastp v0.23.4 (*14*) and mapped the filtered reads to the SARS-CoV-2 reference genome MN908947.3 with BWA-MEM v 0.7.17 (Li H, unpub. data, http://arxiv.org/abs/1303.3997). We used reference genome MN908947.3 when some positions problematic for variant calling were masked as recommended for ARTIC 4.1 protocol (https://github.com/joshquick/artic-ncov2019/tree/master/primer_schemes/nCoV-2019/V4.1). We used iVar v1.4.2 to trim primers and generate final fasta sequences (*15*).

### Virus Isolation

We shipped the SARS-CoV-2–positive nasal swab specimens to St. Jude Children’s Research Hospital (Memphis, TN, USA), where virus isolation, characterization, and in vivo experiments were performed under Biosafety Level 3 laboratory and animal Biosafety Level 3+ conditions. We received Vero E6 cells expressing both TMPRSS2 and ACE2 genes (VE6A2T2; kindly provided by Dr. Barney Graham at the National Institute of Allergy and Infectious Diseases). We washed confluent cultures with sterile phosphate buffered saline and overlaid with 100 μL of swab suspension plus 900 μL of infection media (Dulbecco Modified Eagle Medium supplemented with 2% heat treated fetal bovine serum and 1× antimicrobial solution) for 1 hour to enable virus adsorption. We aspirated the inoculum and added fresh infection media to the cells. We checked the cells daily for cytopathic effect and harvested cultures reaching >90% cytopathic effect. We then subjected negative cultures from swab specimens previously identified as Alpha lineage SARS-CoV 2 to a second, undiluted blind passage.

We attempted virus isolation on all Alpha lineage swab specimens (n = 10) by first passing VTM through a 32-mm 0.8/0.2 μM syringe filter. We anesthetized 8–10-week male lakeview golden Syrian hamsters (Charles River Laboratories, https://www.criver.com) with 4% isoflurane and inoculated intranasally with 100 μL of filtered VTM (n = 1/swab). We monitored the hamsters daily. On 2 and 6 days postinfection, we anesthetized the animals with 100 mg/kg ketamine and washed nasal passages with 0.5 mL phosphate buffered saline. All animals were humanely euthanized after the second postinfection nasal wash, and we collected lung and nasal turbinate samples that were homogenized in 5× volume of media. We inoculated supernatants from nasal washes and tissue homogenates onto VE6A2T2 cell cultures for virus isolation. Animal studies were approved by St. Jude Children’s Research Hospital’s Animal Care and Use Committee (protocol no. 442).

### Phylogenetic Analysis

We used the pangolin tppl (*16*) to determine the SARS-CoV-2 lineage for the 36 viruses sequenced for this study. On the basis of those results, we divided the data into 2 datasets, 1 for the Alpha variant (SARS-CoV-2 B.1.1.7) and 1 for the Omicron variant, which included 5 PANGO SARS-CoV-2 lineages (BQ.1.1, BQ.1.1.63, BQ.1.1.67, BQ.1.23, and XBB.1.5.35). We downloaded separate background datasets from GISAID (https://www.gisaid.org) for Alpha and Omicron. The date range for the Omicron sequences was November 1, 2022–March 31, 2023. For the Alpha background dataset, we used the same dataset in our previously published study of Alpha viruses circulating in WTD in Ohio (*11*), with the addition of more recently published sequences from GISAID from humans, deer, and mink. Although the large human dataset was randomly subsampled, all sequences from deer and mink were retained. We aligned the 2 datasets (Alpha and Omicron) by using NextClade (version 3.13.3, https://clades.nextstrain.org) with the wild-type SARS-CoV-2 as a reference. We used in-house Python scripts to remove noncoding regions and mask sites that are known to be unreliable. We inferred phylogenetic trees by using maximum-likelihood methods available in IQ-TREE version 1.6.12 (*17*) with a general time reversible with unequal rates and unequal base frequency plus discrete gamma model of nucleotide substitution and 1,000 bootstrap replicates, by using the high-performance computational capabilities of the Biowulf Linux cluster at the National Institutes of Health (http://biowulf.nih.gov). We visualized the inferred tree by using FigTree v.1.4.4 (https://github.com/rambaut/figtree/releases). We defined deer transmission clusters by monophyletic groups of WTD viruses supported by high bootstrap values (>70) and confirmed or refined by using ultrafast sample placement on existing trees (*18*). None of the deer transmission clusters involved genetically identical viruses, which would be the case if the deer had acquired the virus from a common environmental source such as water.

We reconstructed mutations on the phylogenetic tree by using TreeTime v0.11.4 (*19*). We reconstructed root-to-tip regression and mutation contexts with in-house R scripts in R (The R Project for Statistical Computing, https://www.r-project.org), as described previously (*11*). We visualized the spike glycoprotein mutations with ChimeraX (*20*) and visualized mutations in B.1.1.7 deer clusters with the ete3 package in Python (*21*).

### Bayesian Analysis

We examined the evolutionary relationships between humans and WTD in greater detail for the Alpha variant. The analysis included an additional 17 genome sequences from B.1.1.7 viruses collected from WTD in Pennsylvania during 2022–2023 that were provided by the US Department of Agriculture (USDA). We contacted the submitting authors of all B.1.1.7 sequences collected from humans in 2022–2023 and published on GISAID. Those communications resulted in identifying some GISAID entries as mislabeled; those entries were subsequently corrected and, in rare cases, confirmed as chronically infected patients. We retained all B.1.1.7 sequences with virus collection dates during 2022–2023 confirmed by submitting authors to have the correct date for our analysis. We performed a time-scaled Bayesian analysis by using the Markov chain Monte Carlo method available by using the latest version of the BEAST (*22*) package with graphics processing units available from the Biowulf Linux cluster. We used a host-specific local clock (*23*) to accommodate differences in the evolutionary rate between WTD and humans. Because WTD viruses were not monophyletic on the Alpha or Delta tree, owing to multiple independent human-to-deer transmission events, we identified separate WTD transmission clusters on the maximum-likelihood tree. We used a Bayesian nonparametric demographic model (*24*) with a general time reversible model of nucleotide substitution with gamma-distributed rate variation among sites. We ran the Markov chain Monte Carlo separately 3–5 times for each dataset by using the BEAGLE 3 library (*25*) to improve computational performance, until all parameters reached convergence as assessed visually by using Tracer v.1.7.2 (*26*). We removed >10% of the chain as burn-in and combined runs for the same dataset by using LogCombiner v1.10.4 (https://beast.community/logcombiner). We summarized a maximum clade credibility tree by using TreeAnnotator version 1.10.4 (https://beast.community/treeannotator). We conducted a phylogeographic discrete trait analysis (*27*) to quantify rates of viral gene flow, particularly in the directions of human-to-deer and long-distance deer-to-deer transmission. We specified a location state for each viral sequence. The 5 categories were B.1.1.7 viruses collected in humans; B.1.1.7 viruses collected in WTD in Ohio, including northern Ohio sequences generated for this study and sequences from southern Ohio generated for our previous study; B.1.1.7 viruses collected in WTD in Pennsylvania during 2022–2023 that were provided by USDA; all other B.1.1.7 viruses collected in WTD; and B.1.1.7 viruses collected from mink in Europe (including Poland and Lithuania). We estimated the expected number of location state transitions in the ancestral history on the basis of the data observed from the tree tips by using Markov jump counts (*28*,*29*), which provided a quantitative measure of asymmetry in gene flow between defined populations.

## Results

### High Prevalence of SARS-CoV-2 in WTD in Northeast Ohio

**Figure 1:**
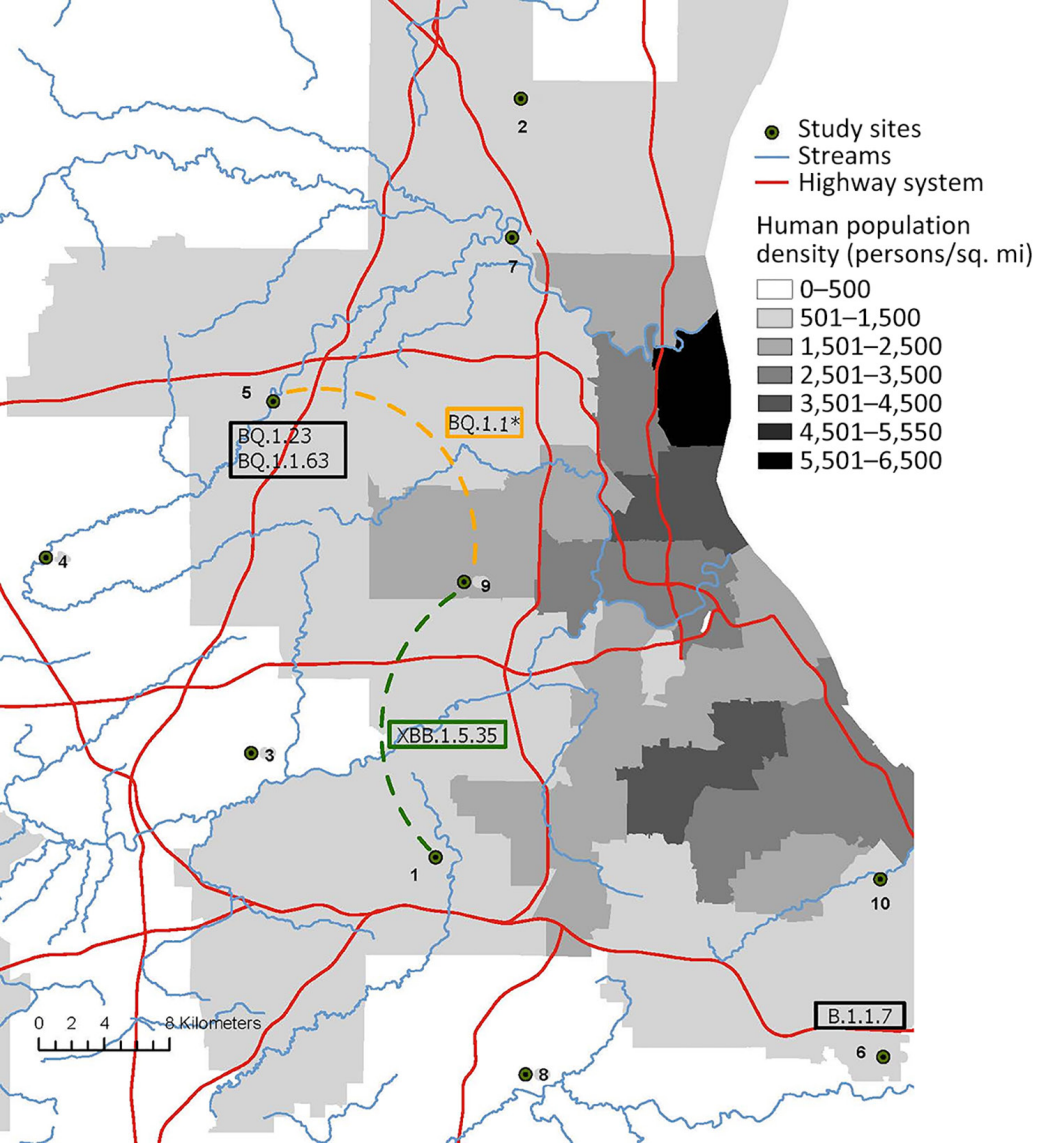
Locations of SARS-CoV-2 viruses in white-tailed deer in northeast Ohio, USA. The map shows the 6 pangolin lineages (*17*) identified in deer (boxes) located within 4 sampling sites during January–March 2023. The dotted lines between sampling sites represent inferred routes of transmission, as inferred from deer clusters identified on the phylogenetic tree shown in Figure 2. The SARS-CoV-2 XBB.1.5.35 lineage transmission between locations 1 and 9 is inferred from Omicron cluster 1. The SARS-CoV-2 BQ.1.1 (including BQ.1.1.67) lineage transmission between locations 5 and 9 is inferred from Omicron cluster 2. Map created by using ArcGIS Pro 3.1.0 (Esri, https://www.esri.com).

Of nasal swab samples collected from WTD in northeast Ohio during January–March 2023, a total of 12.3% (64/519) were positive for SARS-CoV-2 by RT-PCR (Appendix Table 1, Figure 1). SARS-CoV-2 was detected in 4 of the 10 study sites, ranging in estimated prevalence of 6.5%–50.0%. We estimated the highest prevalence (50.0%, 95% CI 33.38%–66.62%) at site 9, a location adjacent to an area of high human population density (Figure 1). We attempted virus isolation on 58 of the 64 SARS-CoV-2 RT-PCR–positive samples, yielding 12 SARS-CoV-2 isolates, all belonging to the Omicron lineage (Appendix Table 2).

### Alpha and Omicron Variants Identified in WTD

We generated whole-genome sequences from 36 SARS-CoV-2 RT-PCR–positive samples collected from WTD in northeast Ohio during January 25–March 13, 2023. Whole-genome SARS-CoV-2 sequences are available in GenBank (Appendix Table 3). Most viruses (86.1%, 31/36) were classified as PANGO lineages belonging to the Omicron variant (BQ.1.1, BQ.1.1.63, BQ.1.1.67, BQ.1.23, and XBB.1.5.35) (Table) that was circulating in humans at the time in Ohio and globally. However, 5 viruses collected from WTD in this study were classified as PANGO lineage B.1.1.7 (Alpha variant). B.1.1.7 viruses caused an outbreak in humans in Ohio during the spring of 2021, almost 2 years before our sampling period in deer. The last B.1.1.7 virus sampled in humans in Ohio was on August 23, 2021 (GISAID accession no. EPI_ISL_3897556). All 5 B.1.1.7 viruses identified in WTD from this study were sampled at the same location (region 6) (Figure 1) on the same date (January 26, 2023). The most closely related human B.1.1.7 virus was collected >1 year earlier on November 19, 2021 (strain identification no. hCoV-19/USA/DC-DFS-PHL-03511/2021).

**Table:** SARS-CoV-2 whole genome sequences generated for a study on the persistence of SARS-CoV-2 Alpha variant in white-tailed deer, Ohio, USA

Strain name	Region	Pangolin lineage (*17*)	Date	Cluster
SC2/deer/USA/OH-OSU-COV0045057/2023	R6	B.1.1.7	2023 Jan 26	Alpha cluster
SC2/deer/USA/OH-OSU-COV0045054/2023	R6	B.1.1.7	2023 Jan 26	Alpha cluster
SC2/deer/USA/OH-OSU-COV0045056/2023	R6	B.1.1.7	2023 Jan 26	Alpha cluster
SC2/deer/USA/OH-OSU-COV0045058/2023	R6	B.1.1.7	2023 Jan 26	Alpha cluster
SC2/deer/USA/OH-OSU-COV0045967/2023	R6	B.1.1.7	2023 Jan 26	Alpha cluster
SC2/deer/USA/OH-OSU-COV0054305/2023	R1	XBB.1.5.35	2023 Feb 23	Omicron cluster 1
SC2/deer/USA/OH-OSU-COV0054309/2023	R1	XBB.1.5.35	2023 Feb 23	Omicron cluster 1
SC2/deer/USA/OH-OSU-COV0054308/2023	R1	XBB.1.5.35	2023 Feb 23	Omicron cluster 1
SC2/deer/USA/OH-OSU-COV0054313/2023	R1	XBB.1.5.35	2023 Feb 23	Omicron cluster 1
SC2/deer/USA/OH-OSU-COV0054303/2023	R1	XBB.1.5.35	2023 Feb 23	Omicron cluster 1
SC2/deer/USA/OH-OSU-COV0054306/2023	R1	XBB.1.5.35	2023 Feb 23	Omicron cluster 1
SC2/deer/USA/OH-OSU-COV0054343/2023	R9	XBB.1.5.35	2023 Feb 27	Omicron cluster 1
SC2/deer/USA/OH-OSU-COV0054342/2023	R9	XBB.1.5.35	2023 Feb 27	Omicron cluster 1
SC2/deer/USA/OH-OSU-COV0060785/2023	R1	XBB.1.5.35	2023 Mar 9	Omicron cluster 1
SC2/deer/USA/OH-OSU-COV0060778/2023	R1	XBB.1.5.35	2023 Mar 9	Omicron cluster 1
SC2/deer/USA/OH-OSU-COV0060777/2023	R1	XBB.1.5.35	2023 Mar 9	Omicron cluster 1
SC2/deer/USA/OH-OSU-COV0060782/2023	R1	XBB.1.5.35	2023 Mar 9	Omicron cluster 1
SC2/deer/USA/OH-OSU-COV0060781/2023	R1	XBB.1.5.35	2023 Mar 9	Omicron cluster 1
SC2/deer/USA/OH-OSU-COV0060793/2023	R1	XBB.1.5.35	2023 Mar 9	Omicron cluster 1
SC2/deer/USA/OH-OSU-COV0045880/2023	R9	BQ.1.1	2023 Jan 30	Omicron cluster 2
SC2/deer/USA/OH-OSU-COV0045866/2023	R9	BQ.1.1	2023 Jan 30	Omicron cluster 2
SC2/deer/USA/OH-OSU-COV0045876/2023	R9	BQ.1.1	2023 Jan 30	Omicron cluster 2
SC2/deer/USA/OH-OSU-COV0045889/2023	R9	BQ.1.1	2023 Jan 30	Omicron cluster 2
SC2/deer/USA/OH-OSU-COV0045870/2023	R9	BQ.1.1	2023 Jan 30	Omicron cluster 2
SC2/deer/USA/OH-OSU-COV0045877/2023	R9	BQ.1.1	2023 Jan 30	Omicron cluster 2
SC2/deer/USA/OH-OSU-COV0045873/2023	R9	BQ.1.1.67	2023 Jan 30	Omicron cluster 2
SC2/deer/USA/OH-OSU-COV0045874/2023	R9	BQ.1.1.67	2023 Jan 30	Omicron cluster 2
SC2/deer/USA/OH-OSU-COV0045887/2023	R9	BQ.1.1	2023 Jan 30	Omicron cluster 2
SC2/deer/USA/OH-OSU-COV0045890/2023	R9	BQ.1.1	2023 Jan 30	Omicron cluster 2
SC2/deer/USA/OH-OSU-COV0054341/2023	R9	BQ.1.1.67	2023 Feb 27	Omicron cluster 2
SC2/deer/USA/OH-OSU-COV0054302/2023	R9	BQ.1.1	2023 Feb 27	Omicron cluster 2
SC2/deer/USA/OH-OSU-COV0060771/2023	R5	BQ.1.1	2023 Mar 13	Omicron cluster 2
SC2/deer/USA/OH-OSU-COV0060768/2023	R5	BQ.1.1	2023 Mar 13	Omicron cluster 2
SC2/deer/USA/OH-OSU-COV0060769/2023	R5	BQ.1.1	2023 Mar 13	Omicron cluster 2
SC2/deer/USA/OH-OSU-COV0045465/2023	R5	BQ.1.23	2023 Jan 25	Omicron singleton 1
SC2/deer/USA/OH-OSU-COV0054384/2023	R5	BQ.1.1.63	2023 Feb 13	Omicron singleton 2

### Deer-to-Deer Transmission between Locations

**Figure 2:**
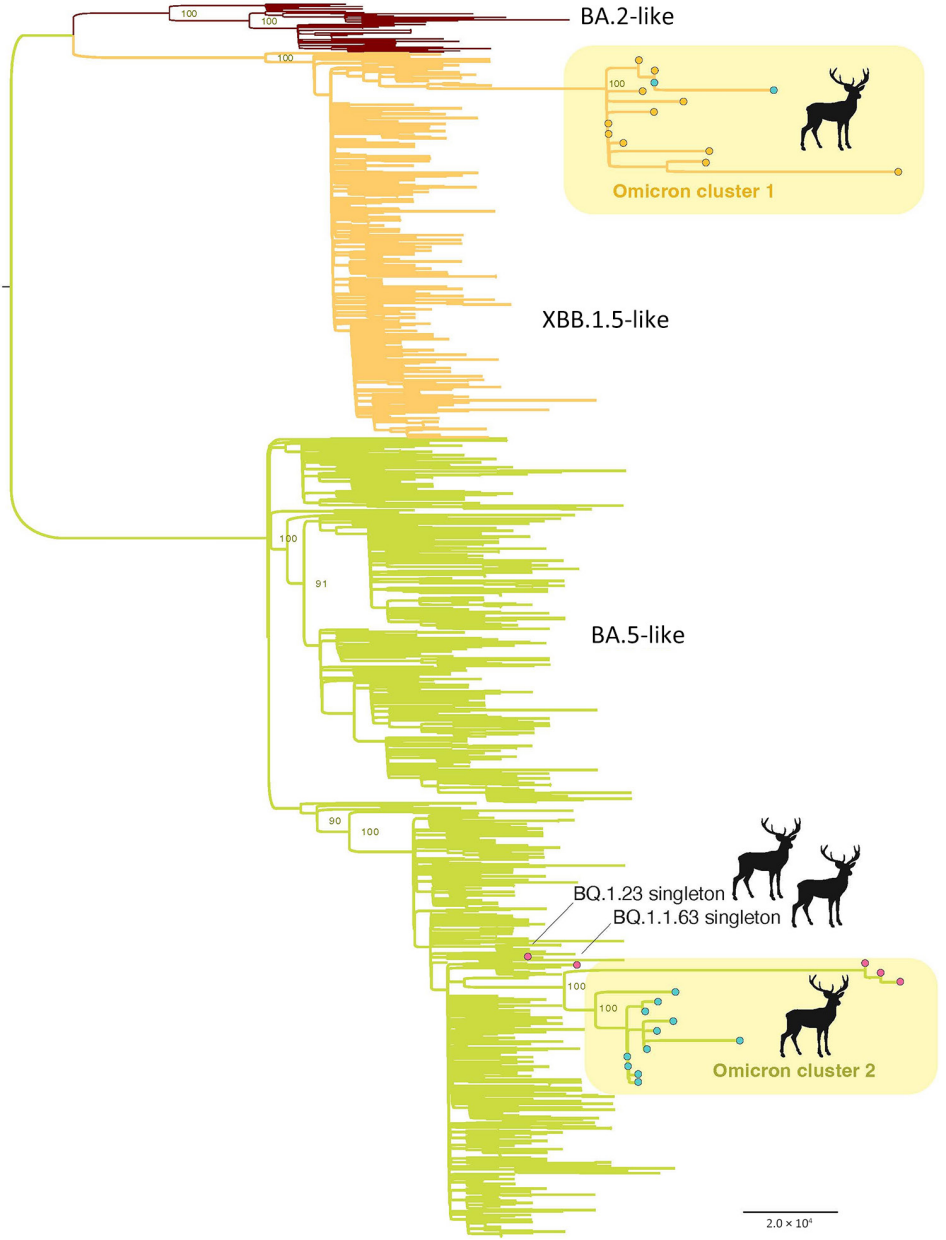
Maximum-likelihood tree inferred for the complete genome sequences of 31 SARS-CoV-2 viruses collected from white-tailed deer and a random subsample of 1,000 SARS-CoV-2 viruses collected from humans from northeast Ohio, November 1, 2022–March 30, 2023. Branches are shaded according to 3 categories of Omicron viruses: red, BA.2-like; orange, XBB.1.5-like; and green, BA.5-like. Circles at tips indicate the deer viruses and are shaded according to region: orange, R1; pink, R5; and blue, R9. Two deer clusters (Omicron cluster 1 and 2) are labeled, along with the 2 single deer singleton viruses. All branch lengths are drawn to scale and bootstrap values are provided for key nodes. Scale bar represents substitutions per site.

Two major deer clusters of Omicron viruses were evident on the phylogenetic tree (Table; Figure 2). Omicron cluster 1 includes 14 viruses belonging to the XBB.1.5.35 lineage sampled at the region 1 (n = 12) and region 9 (n = 2) locations (Figure 1) during February 23–March 9, 2023. Omicron cluster 2 includes 15 viruses belonging to the BQ.1.1 lineage and closely related BQ.1.1.67 lineage and was sampled in the region 5 (n = 3) and region 9 (n = 12) locations (Figure 1) during January 30–March 13, 2023. The presence of 2 clusters spanning multiple locations separated by >10 km is consistent with substantial deer-to-deer transmission of Omicron viruses. No BQ.1.1 or XBB.1.5.35 viruses were observed in any other nonhuman population outside this study on the basis of the sequences available in GISAID.

### Persistence of the Alpha Variant in WTD

**Figure 3:**
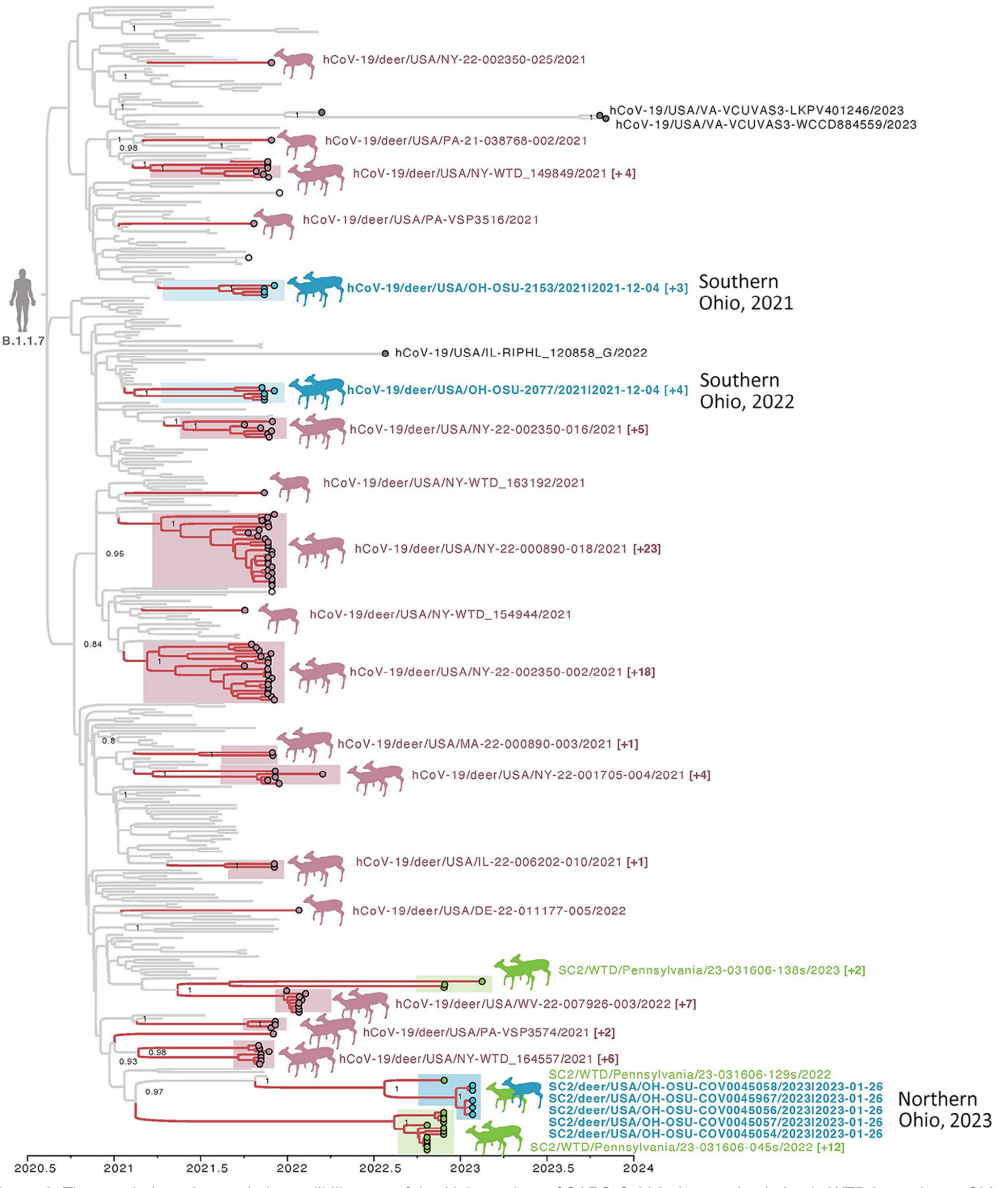
Time-scaled maximum clade credibility tree of the Alpha variant of SARS-CoV-2 viruses circulating in WTD in northeast Ohio, USA. The tree was inferred by using a host-specific local clock for the complete genome sequences of 362 SARS-CoV-2 clade B.1.1.7 viruses sampled December 29, 2020–November 3, 2023. Branches are shaded by host: gray, human or mink; pink, WTD. Circles at tips are shaded by location and source: blue, WTD from Ohio; green, WTD from Pennsylvania (2022–2023); pink, WTD from other states (or Pennsylvania in 2021); white, mink (Lithuania and Poland); and gray, human (2022–2023). Deer transmission clusters are shaded similarly and indicated by paired deer illustrations. Single-deer illustrations indicate singleton introductions into WTD. Singleton WTD viruses are labeled, and a representative virus is provided for each WTD transmission cluster, with the number of additional viruses in the cluster in parentheses. Posterior probabilities are provided for key nodes. WTD, white-tailed deer.

Because B.1.1.7 viruses were reported previously in WTD in Ohio, New York, Pennsylvania, and other US states, as well as in mink in Europe (Poland and Lithuania), we performed an expansive global analysis including B.1.1.7 sequences from humans, deer, and mink that were downloaded from GISAID. We also included 17 additional B.1.1.7 sequences provided by the USDA from WTD in Pennsylvania, which borders northeast Ohio. The maximum clade credibility tree inferred from this dataset (Figure 3) reveals that B.1.1.7 viruses repeatedly transmitted from humans to WTD in New York (9 introductions), Pennsylvania (6 introductions), Ohio (3 introductions), Massachusetts (1 introduction), Delaware (1 introduction), Illinois (1 introduction), and West Virginia (1 introduction). As expected, most suspected human-to-deer B.1.1.7 transmission events occurred during spring 2021, when the Alpha variant was peaking in humans in the United States. Nearly three quarters of B.1.1.7 viruses in WTD were collected in 2021 (73.5%, n = 89/121). A small number of B.1.1.7 viruses were still found in WTD during January 1–March 31, 2022 (8.3%, n = 10/121). However, our sampling in northeast Ohio in 2023 and USDA’s sampling in Pennsylvania occurred a year later, during October 2022–March 2023, and still found 3 clades of B.1.1.7 viruses circulating in WTD in Ohio and Pennsylvania (Figure 3). Our sampling occurred ≈1 year after the virus was no longer identified in WTD in other states. A small number of B.1.1.7 viruses were identified in humans in other US states during 2022–2023 that were collected from a confirmed chronically infected patient (strain identification no. hCoV-19/USA/IL-RIPHL_120858_G/2022 from July 26, 2022 [A. Kittner, Chicago Department of Public Health, pers. comm., email, 2024 Jan 1]) or a suspected chronically infected patient (strain identification no. hCoV-19/USA/VA-VCUVAS3-WCCD884559/2023). We found no other B.1.1.7 sequences in humans globally during 2022–2023, after excluding sequences on GISAID that were determined to be mislabeled, as verified by direct correspondence with the sequence submitters. Of note, the B.1.1.7 viruses collected in deer are not closely related to the viruses sequenced from chronically infected patients (or suspected chronic infection) in Virginia or Illinois, suggesting that deer did not acquire B.1.1.7 from the chronically infected patients for which we have sequence data.

Most Pennsylvania deer B.1.1.7 viruses from 2022–2023 cluster separately from the Ohio deer B.1.1.7 viruses collected in 2023 and represent independent introductions from humans to WTD in Pennsylvania (Figure 3). However, 1 Pennsylvania virus collected from WTD on November 27, 2022 (strain identification no. SC2/WTD/Pennsylvania/23-031606-129s/2022), clusters with the 5 B.1.1.7 Ohio viruses collected in WTD 2 months later, forming a distinct clade that appears to be a deer-to-deer transmission cluster that spans Pennsylvania and Ohio. The Pennsylvania virus was collected in a county situated in the northwest region of the state, bordering northeast Ohio. The maximum clade credibility tree estimates that the human-to-deer transmission event that led to the Ohio/Pennsylvania deer transmission cluster occurred during October 26, 2021–July 23, 2022. We suspect transmission occurred closer to the 2021 end of this range, given that B.1.1.7 was more prevalent in humans in 2021 than 2022. The maximum clade credibility tree also shows that the B.1.1.7 viruses found in WTD from northeast Ohio in 2023 are not closely related to the B.1.1.7 viruses that were found in WTD from southern Ohio in 2021 from our previous study (*11*) and represent a third independent introduction of B.1.1.7 from humans into WTD in Ohio. Whether B.1.1.7 was first introduced from humans into WTD in Ohio and then spread to deer in Pennsylvania, or vice versa, is difficult to determine because of the long branch length and ≈1-year time gap between the estimated human-to-deer introduction and first detection of the cluster in deer. SARS-CoV-2 detections in WTD in Ohio were limited near the end of 2022, and no sequences were recovered from positive samples despite multiple attempts, contributing to uncertainties around transmission and persistence in deer. The most closely related human virus was sampled in the District of Columbia, but the high mobility of humans and long lag time means this data point does not inform our inference of the location of human-to-deer transmission.

### SARS-CoV-2 Mutations in Spike Protein in WTD 

**Figure 4:**
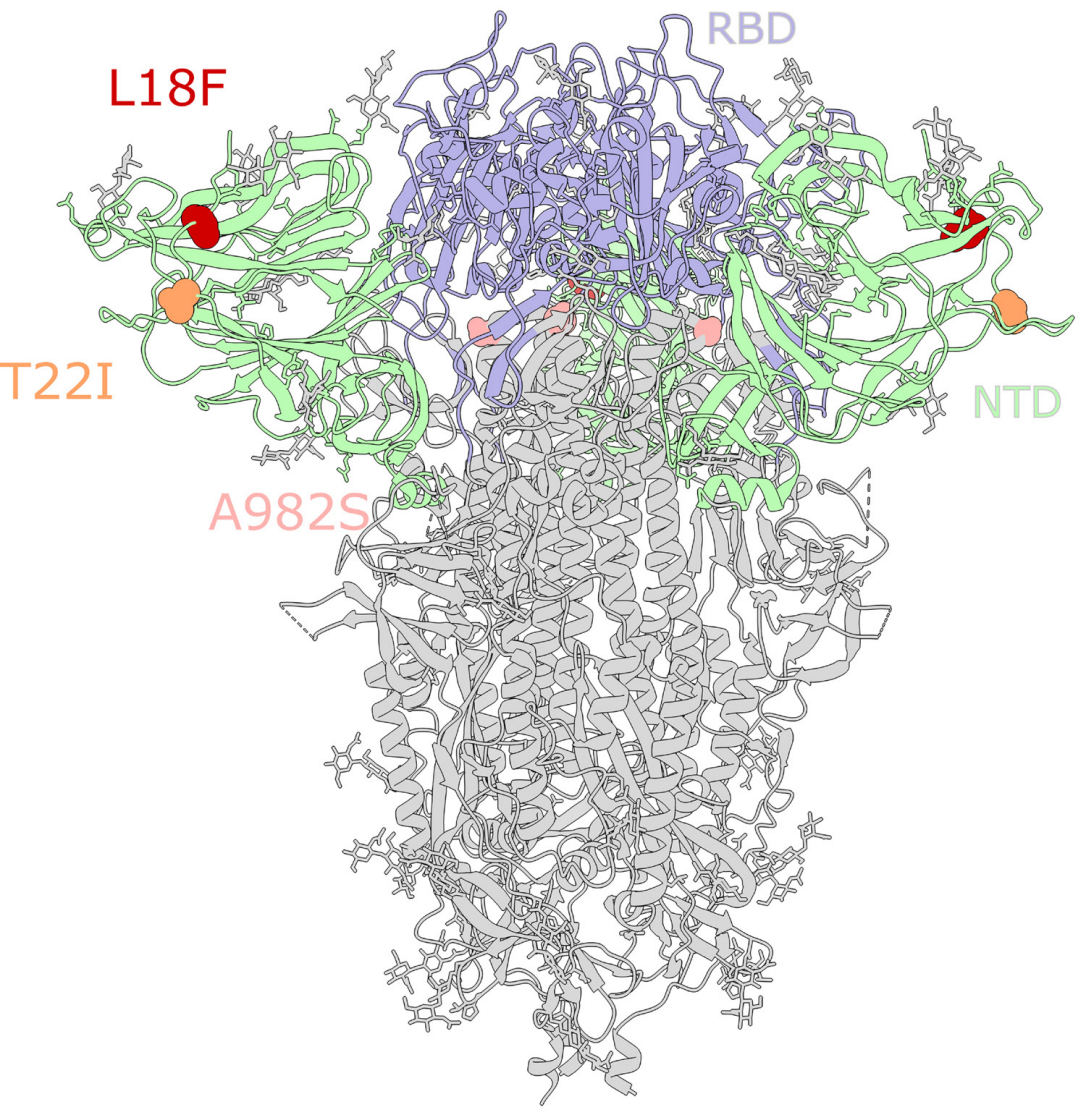
Mutations in the spike glycoprotein of SARS-CoV-2 viruses circulating in white-tailed deer in northeast Ohio, USA. SARS-CoV-2 spike glycoprotein structure obtained from the Protein Data Base (identification no. 7jji; https://www.rcsb.org). RBD and NTD are labeled. Circles indicate the location of mutations in the SARS-CoV-2 clade B.1.1.7 viruses collected from white-tailed deer in Ohio and other US states: red, L18F; orange, T22I; pink, A982S. Mutation S12F (located in NTD) is not shown because it is located at the flexible N terminus, for which a 3D structure is not available. NTD, N-terminal domain; RBD, receptor-binding domain.

 B.1.1.7 viruses in WTD accumulated mutations at a rate that was >2-fold higher than in humans (Appendix Figure 1, panel A). Consistent with our prior findings (*11*), the higher rate of evolution in WTD is not driven by a higher rate of nonsynonymous mutations, which would be evidence of strong selective pressures. Rather, both synonymous and nonsynonymous mutations are accumulated at higher rates (Appendix Figure 1, panels B, C). In particular, T>C substitutions occur more often in deer than in humans (Appendix Figure 1, panels D, E). The presence of multiple independently evolving WTD clusters of B.1.1.7 provides an opportunity to look for recurring mutations that could be adaptive to deer. We observed recurring mutations in the N-terminal domain of the spike protein (S12F, L18F, and T22I) (Figure 4) that each occur independently in 2 different deer clusters (Appendix Figures 1, 2). The L18F mutation was observed in 2021 in WTD in New York and again in 2023 in Pennsylvania. L18F is associated with escape from multiple N-terminal domain binding antibodies in humans (*30*). The 2023 B.1.1.7 viruses from Pennsylvania also contain the A982S reversion mutation in the spike protein. Although S982A was 1 of the characteristic mutations of B.1.1.7, we observed the A982S reversion in 12 samples (3 independent events).

## Discussion

An apparent decrease in SARS-CoV-2 detections in North America WTD after emergence of the Omicron variant in humans in late 2021 led to speculation that the highly mutated and human-adapted Omicron variant could have reduced capacity to infect other species. Instead, we found evidence in northeast Ohio of extensive deer-to-deer transmission of >2 Omicron lineages (XBB.1.5.35 and BQ.1.1) that each spread between 2 sampling sites separated by interstate highways. An even more surprising finding in our study was the detection of 5 Alpha variant B.1.1.7 viruses in WTD in January 2023, more than 1 year after the last detection of B.1.1.7 in humans in Ohio. Additional B.1.1.7 deer transmission clusters were identified in Pennsylvania in late 2022, as well as a cluster in WTD in a neighboring Pennsylvania county that is positioned with our Ohio deer transmission cluster, emphasizing the importance of coordinated surveillance across state lines. Together, those data support the capacity of WTD to sustain Alpha variant transmission after the virus disappeared in humans, even longer than previously reported in WTD in New York and our previous study (*4*,*11*). The retention of SARS-CoV-2 lineages no longer circulating in humans, evolving with an increased mutation rate and accumulation of mutations evading immunity, warrants further monitoring of the persistence and evolution of SARS-CoV-2 in deer. SARS-CoV-2 evolution also provides a rare opportunity to study pathogen emergence and early adaptations in wildlife hosts, before the disease becomes widely established.

A recent report of SARS-CoV-2 spillover from humans to farmed mink to WTD underscores the virus’s high capacity for host-switching (A. Crespo-Bellido, unpub. data). SARS-CoV-2 viruses were also recently detected in a wide range of species in Virginia (*31*). Given the broad host range of SARS-CoV-2, it is possible that the Alpha variant was maintained by multiple host species before detection in WTD in our study, including some that are not sampled, but no detections in other species from this region were reported (*1*). Active surveillance and serosurveys of SARS-CoV-2 in a broad range of species are warranted, along with targeted studies in WTD. Experimental studies also are needed to determine the mode of transmission (e.g., environmental, airborne) between humans and deer and from deer to deer.

More questions than answers remain surrounding the human–animal interface for SARS-CoV-2. Currently, phylogenetic analyses do not suggest the SARS-CoV-2 variants circulating in WTD in northeast Ohio have transmitted back to humans or present a major zoonotic risk, but suspected deer-to-human transmission has been documented in Ontario, Canada (*8*). If SARS-CoV-2 becomes endemic in WTD, the virus may persist with relatively few mutations over time. However, it is also possible the virus could accumulate functional mutations, leading to deer-adapted variants with unknown spillover potential to other hosts. Evidence of sustained transmission of the Alpha variant in WTD, alongside more recent introductions of Omicron lineages in WTD, highlights the need for continued surveillance to monitor the long-term dynamics of SARS-CoV-2 in WTD and the associated zoonotic risks.

Appendix Additional information about persistence of SARS-CoV-2 alpha variant in white-tailed deer, Ohio, USA
