# Supplementary material for: Persistence of SARS-CoV-2 Alpha Variant in White-Tailed Deer, Ohio, USA
Source: Emerg Infect Dis. 2025 Jul;31(7):1319–29. doi: 10.3201/eid3107.241922 (PMC12205454; doi:10.3201/eid3107.241922)
Supplement: Appendix — Additional information about persistence of SARS-CoV-2 alpha variant in white-tailed deer, Ohio, USA [file 24-1922-Techapp-s1.pdf]

Article DOI: https://doi.org/10.3201/eid3107.241922

EID cannot ensure accessibility for supplementary materials supplied by authors. Readers who have difficulty accessing supplementary content should contact the authors for assistance.

Persistence of SARS-CoV-2 Alpha Variant in White-Tailed Deer, Ohio, USA

Appendix

Appendix Table 1. The prevalence of SARS-CoV-2 in white-tailed deer at each study site location was estimated using rRT-PCR results and reported with Clopper-Pearson exact 95% confidence interval*

Region	# Positive Samples	# Total Samples	Prevalence (%)	95% CI
**R1**	21	81	25.9	(16.82, 36.86)
**R2**	0	14	NA	NA
**R3**	0	53	NA	NA
**R4**	0	44	NA	NA
**R5**	7	107	6.5	(2.67, 13.02)
**R6**	17	44	38.6	(24.36, 54.50)
**R7**	0	87	NA	NA
**R8**	0	19	NA	NA
**R9**	19	38	50.0	(33.38, 66.62)
**R10**	0	32	NA	NA
**Total**	64	519	12.3	(9.59, 15.41)

*NA, not available

Appendix Table 2. 12 SARS-CoV-2 viral isolates.

Virus Name	Lineage
SARS-CoV-2/white-tailed deer/USA/OH-OSU-COV0045465 /2023	Omicron BQ.1
SARS-CoV-2/white-tailed deer/USA/OH-OSU-COV0045866 /2023	Omicron BQ1.1
SARS-CoV-2/white-tailed deer/USA/OH-OSU-COV0054308 /2023	Omicron XBB.1.5.35
SARS-CoV-2/white-tailed deer/USA/OH-OSU-COV0013570 /2023	Omicron BF.26
SARS-CoV-2/white-tailed deer/USA/OH-OSU-COV0045189 /2023	Omicron BY.1
SARS-CoV-2/white-tailed deer/USA/OH-OSU-COV0045199 /2023	Omicron BY.1
SARS-CoV-2/white-tailed deer/USA/OH-OSU-COV0045919 /2023	Omicron BF.7.4
SARS-CoV-2/white-tailed deer/USA/OH-OSU-COV0045870 /2023	Omicron BQ1.1
SARS-CoV-2/white-tailed deer/USA/OH-OSU-COV0045876 /2023	Omicron BQ1.1
SARS-CoV-2/white-tailed deer/USA/OH-OSU-COV0045880 /2023	Omicron BQ1.1
SARS-CoV-2/white-tailed deer/USA/OH-OSU-COV0045889 /2023	Omicron BQ1.1
SARS-CoV-2/white-tailed deer/USA/OH-OSU-COV0054302 /2023	Omicron XBB

Appendix Table 3. 36 WGS submitted to GenBank with associated accession numbers.

Virus Name	GenBank Accession Number
SARS-CoV-2/white-tailed deer/USA/OH-OSU-COV0045054/2023	PQ529779
SARS-CoV-2/white-tailed deer/USA/OH-OSU-COV0045056/2023	PQ541515
SARS-CoV-2/white-tailed deer/USA/OH-OSU-COV0045057/2023	PQ529821
SARS-CoV-2/white-tailed deer/USA/OH-OSU-COV0045058/2023	PQ529936
SARS-CoV-2/white-tailed deer/USA/OH-OSU-COV0045465/2023	PQ498325
SARS-CoV-2/white-tailed deer/USA/OH-OSU-COV0045866/2023	PQ536466
SARS-CoV-2/white-tailed deer/USA/OH-OSU-COV0045870/2023	PQ529823
SARS-CoV-2/white-tailed deer/USA/OH-OSU-COV0045873/2023	PQ498261
SARS-CoV-2/white-tailed deer/USA/OH-OSU-COV0045874/2023	PQ529776
SARS-CoV-2/white-tailed deer/USA/OH-OSU-COV0045876/2023	PQ498319
SARS-CoV-2/white-tailed deer/USA/OH-OSU-COV0045877/2023	PQ498321
SARS-CoV-2/white-tailed deer/USA/OH-OSU-COV0045880/2023	PQ498262
SARS-CoV-2/white-tailed deer/USA/OH-OSU-COV0045887/2023	PQ529780
SARS-CoV-2/white-tailed deer/USA/OH-OSU-COV0045889/2023	PQ498258
SARS-CoV-2/white-tailed deer/USA/OH-OSU-COV0045890/2023	PQ498320

Virus Name	GenBank Accession Number
SARS-CoV-2/white-tailed deer/USA/OH-OSU-COV0045967/2023	PQ529824
SARS-CoV-2/white-tailed deer/USA/OH-OSU-COV0054302/2023	PQ609695
SARS-CoV-2/white-tailed deer/USA/OH-OSU-COV0054303/2023	PQ529935
SARS-CoV-2/white-tailed deer/USA/OH-OSU-COV0054305/2023	PQ529820
SARS-CoV-2/white-tailed deer/USA/OH-OSU-COV0054306/2023	PQ529822
SARS-CoV-2/white-tailed deer/USA/OH-OSU-COV0054308/2023	PQ529825
SARS-CoV-2/white-tailed deer/USA/OH-OSU-COV0054309/2023	PQ529937
SARS-CoV-2/white-tailed deer/USA/OH-OSU-COV0054313/2023	PQ541496
SARS-CoV-2/white-tailed deer/USA/OH-OSU-COV0054341/2023	PQ498317
SARS-CoV-2/white-tailed deer/USA/OH-OSU-COV0054342/2023	PQ529777
SARS-CoV-2/white-tailed deer/USA/OH-OSU-COV0054343/2023	PQ529756
SARS-CoV-2/white-tailed deer/USA/OH-OSU-COV0054384/2023	PQ498318
SARS-CoV-2/white-tailed deer/USA/OH-OSU-COV0060768/2023	PQ541133
SARS-CoV-2/white-tailed deer/USA/OH-OSU-COV0060769/2023	PQ498322
SARS-CoV-2/white-tailed deer/USA/OH-OSU-COV0060771/2023	PQ529778
SARS-CoV-2/white-tailed deer/USA/OH-OSU-COV0060777/2023	PQ530036
SARS-CoV-2/white-tailed deer/USA/OH-OSU-COV0060778/2023	PQ541495
SARS-CoV-2/white-tailed deer/USA/OH-OSU-COV0060781/2023	PQ541134
SARS-CoV-2/white-tailed deer/USA/OH-OSU-COV0060782/2023	PQ529934
SARS-CoV-2/white-tailed deer/USA/OH-OSU-COV0060785/2023	PQ529938
SARS-CoV-2/white-tailed deer/USA/OH-OSU-COV0060793/2023	PQ529933

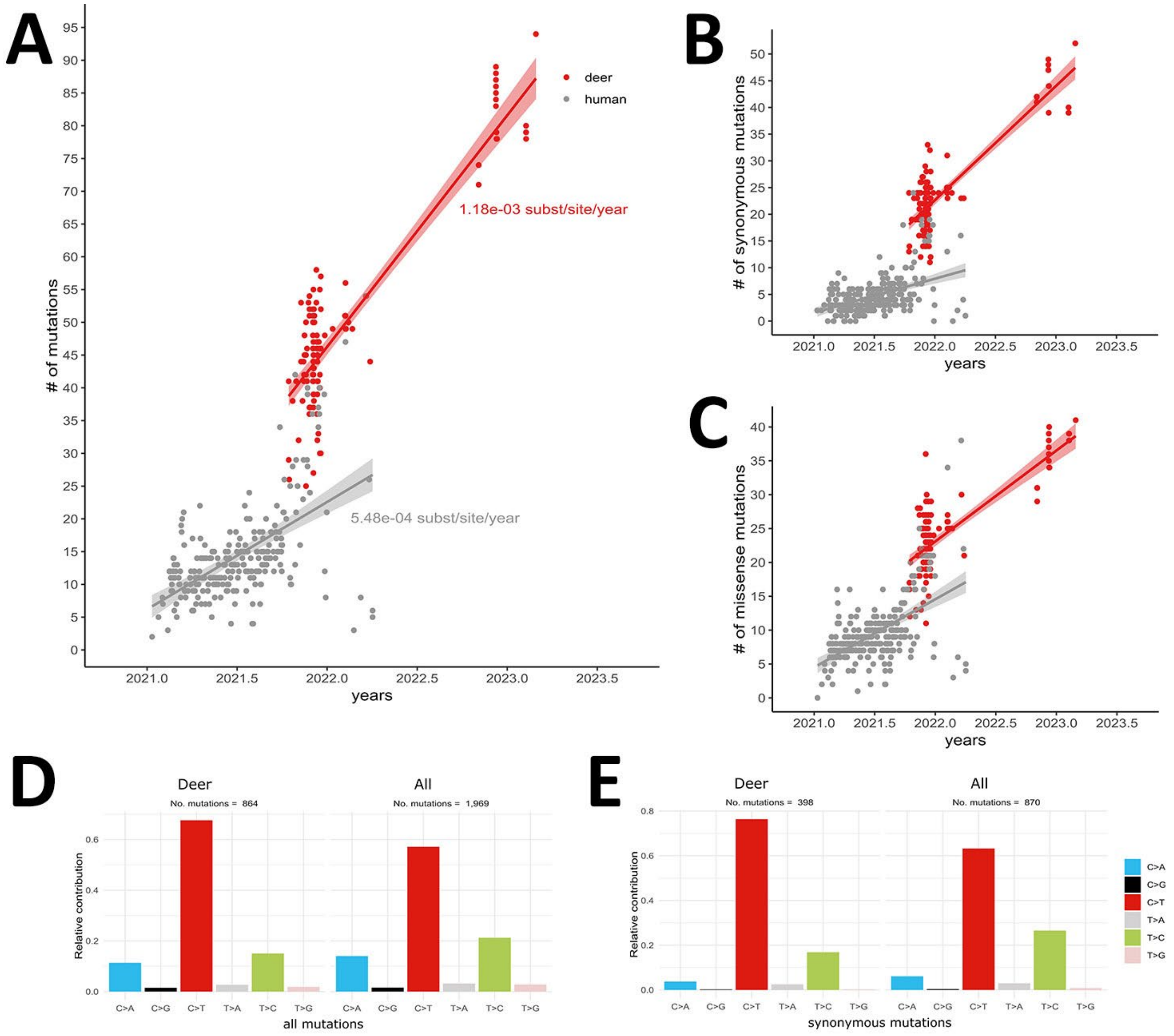

Appendix Figure 1. Mutations in deer B.1.1.7 viruses. Regression lines for the number of accumulated mutations in deer (red) vs human (grey) (a) for all mutations (b) only for synonymous mutations (c) for missense mutations. Prevalence of T>C substitutions in deer (d) all mutations, (e) only synonymous mutations.

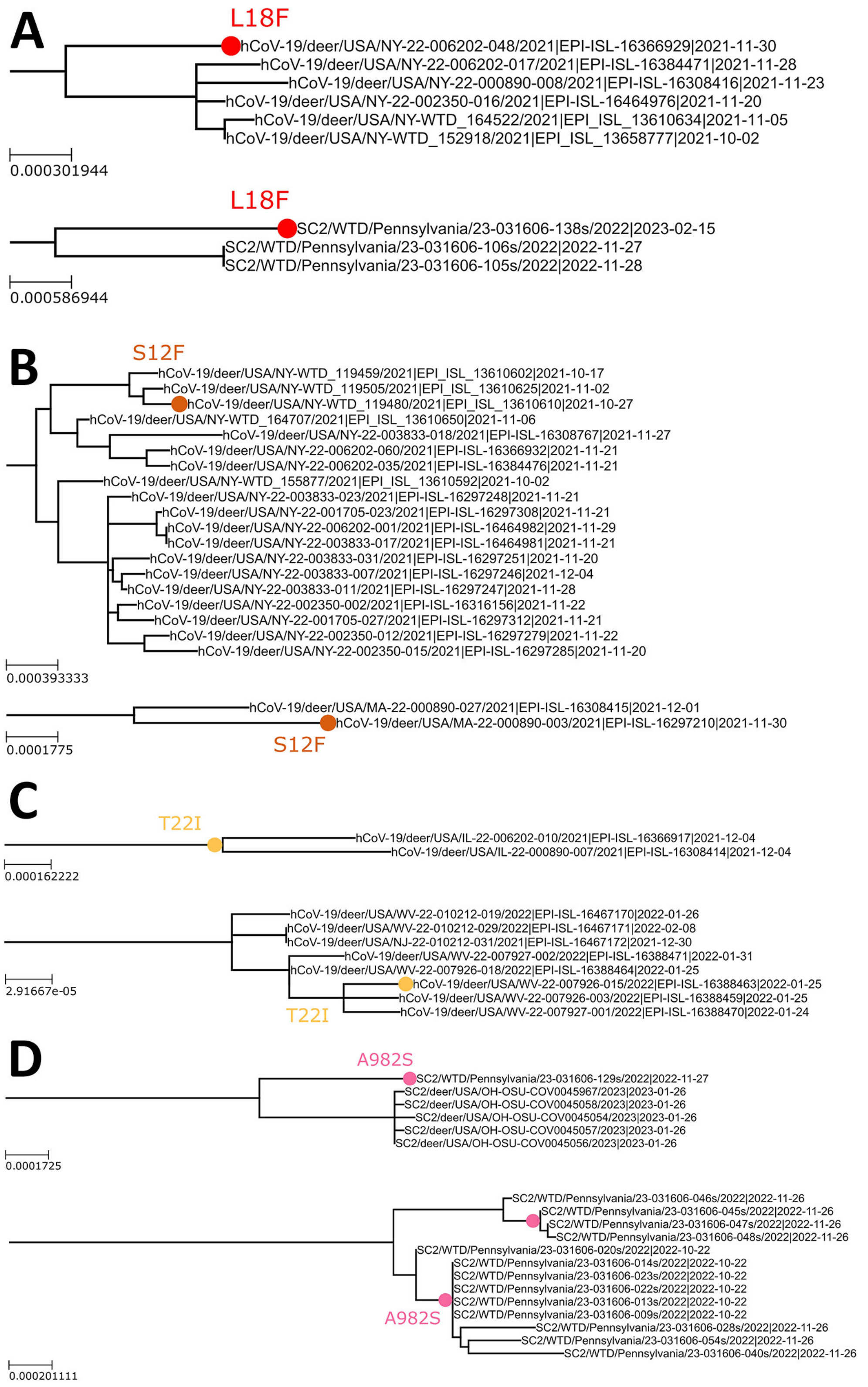

Appendix Figure 2. Mutations in spike protein in WTD. Distribution of L18F (a), S12F (b), T22I (c), and A982S (d). Nodes where mutations occur are marked with colored circles.
